# Anti-oxidant, anti-inflammatory and antimicrobial activity of aqueous extract from acerola and amla

**DOI:** 10.6026/973206300200765

**Published:** 2024-07-31

**Authors:** Chellathurai Burnice Nalina Kumari, Namasivayam Ambalavanan, Shanmugam Rajesh Kumar, Jaideep Mahendra, Uma Sudhakar

**Affiliations:** 1 Department of Periodontics, Meenakshi Ammal Dental College and Hospital, Meenakshi Academy of Higher Education and Research, Chennai, Tamil Nadu, India; 2 Nano biomedicine Lab, Centre for Global Health Research, Saveetha Institute of Medical and Technical Sciences, Chennai, Tamil Nadu, India; 3 Department of Periodontics, Thai Moogambigai Dental College and Hospital, Tamil Nadu, India

**Keywords:** Amla extract, Acerola extract, Antioxidant agent, Anti-inflammatory agent, Antibacterial agent, Biocompatibility

## Abstract

Amla, scientifically known as *emblica officinalis* and Acerola (*malphigian emarginata*) both are Vitamin C fruits possess varied medicinal properties being used for preventive disease health management strategies. Therefore, it is of interest to explore the antioxidant, anti-inflammatory, antibacterial, and cytotoxic properties of aqueous extracts from Acerola and Amla. Hence, the anti-inflammatory activity of Acerola and amla was assessed using the bovine serum albumin denaturation assay (BSA Assay), antioxidant properties were compared using DPPH (2,2-diphenyl-1-picrylhydrazyl) assay. Both extracts antibacterial activities were evaluated through the agar well diffusion technique against oral pathogens and Brine shrimp lethality assay for cytotoxicity. The current research sheds light on natural remedies for oxidative stress-related diseases, inflammatory conditions and bacterial infections, offering promising avenues for disease management and preventive healthcare strategies especially in the treatment of oral health diseases like periodontitis.

## Background:

Oral diseases attributed to pathogens have emerged as a significant concern in recent years. The identification of Severe Acute Respiratory Syndrome Coronavirus-2 (SARS-CoV-2) in the oral cavity suggests a potential impact on oral health [1]. Moreover, there is a documented association between periodontal pathogens and the onset of oral cancer, underscoring a link between periodontal diseases and various human cancers [2]. Candida, recognized as an opportunistic pathogen, is implicated in oral conditions like oral candidiasis and denture stomatitis, as well as systemic ailments such as aspiration pneumonia and fungemia [3]. The presence of oral pathogens, especially in biofilms, is a contributing factor to the development of dental caries and periodontal disease, with conventional therapeutic approaches showing diminishing effectiveness due to increased drug resistance [4]. The immune response within the oral cavity plays a pivotal role in averting oral fungal infections, and the interactions with other members of the oral microbiome can influence microbial pathogenicity [5]. Numerous studies have extensively explored the therapeutic potential of herbal extracts in the treatment of oral diseases and such extracts obtained from medicinal herbs and plants exhibit notable therapeutic actions, including anti-inflammatory, antimicrobial, and immune system regulatory properties, rendering them attractive alternatives to synthetic drugs within oral cavity [6, 7]. The rising popularity of herbal mouthwashes can be attributed to their efficacy against oral pathogens and minimal side effects [8]. Herbal remedies have demonstrated the ability to regulate the production of proinflammatory mediators, establishing them as safer alternatives to chemical anti-inflammatory drugs [9]. Various herbs have been recognized as viable alternatives for managing oral conditions such as caries prevention, gingivitis, periodontitis, oral ulcers, and inflammation [10]. Amla, scientifically known as *Phyllanthus emblica*, holds substantial medicinal importance in the Unani system of medicine [11]. This plant with active components Phenolic compounds, amino acids, tannins, alkaloids, vitamins and carbohydrates is renowned for its therapeutic effects on heart and brain health, and it has been traditionally employed in the treatment of diverse conditions such as cancer, diabetes, liver diseases, and gastric ulcers [12]. Amla extract has been identified for its antibacterial properties against oral pathogens, coupled with antioxidant and anti-inflammatory attributes. Research indicates that the methanolic extract of amla displays noteworthy antibacterial efficacy against oral pathogens like *Streptococcus mutans*, *Streptococcus oralis*, and *Streptococcus rattus*, commonly associated with oral infections [13]. Moreover, amla extracts exhibit robust antioxidant activity, demonstrated by their capacity to neutralize free radicals and alleviate oxidative stress [14]. Additionally, *In vitro* assays measuring albumin denaturation and 15-lipoxygenase inhibition have revealed the anti-inflammatory effects of amla extracts [15, 16]. Acerola, scientifically known as *Malpighia emarginata*, is a tropical fruit renowned for its high vitamin C content and a spectrum of bioactive compounds, positioning it as a valuable source of nutrition with potential health benefits and given its perishable nature, proper postharvest handling is deemed essential to preserve the fruit's quality [17]. Despite its nutritional value and potential health benefits, acerola remains underutilized in various parts of the world, making it a somewhat overlooked functional super fruit [18]. Notably, Brazil has emerged as a leading producer of acerola, incorporating the fruit into various products such as juices, jams, and sweets [19]. The acerola extract encompasses a diverse array of phenolic compounds, such as quercetin, p-coumaric acid, gallic acid, epigallocatechin gallate, catechin, syringic acid, and epicatechin. These compounds play a significant role in conferring antioxidant and antibacterial activities to the extract [20]. Therefore, it is of interest to explore the antioxidant, anti-inflammatory, antibacterial, and cytotoxic properties of aqueous extracts from Acerola and Amla.

## Materials and Methods:

*In vitro* study, Chennai, conducted between May to July 2023, Amla and Acerola powder (Brut appett) freezed dried powder obtained from RMCA Ventures, Bangalore were used for this study.

## Preparation of plant extract:

A total of 1g of amla powder and 1g of acerola powder were individually weighed and then dissolved in 100mL of distilled water. Each solution was subjected to heat using a heating mantle, maintaining a temperature range of 60-70 degrees Celsius, for duration of 15-20 minutes. Subsequently, the heated solutions were separately filtered using Whatman No:1 filter paper to eliminate any solid residues. The resulting filtered extracts from amla and acerola were then individually condensed to a final volume of 5mL under the same temperature conditions. The concentrated extracts obtained from the amla and acerola powders were utilized for subsequent biomedical applications testing.

## Antioxidant activity:

The DPPH (2,2-diphenyl-1-picrylhydrazyl) assay was conducted to evaluate the antioxidant activity of amla and acerola extracts. A stock solution of 0.1 mM DPPH was initially prepared in methanol. Subsequently, a fresh working solution was created by diluting the stock solution to a final concentration of 20 µM in methanol. Various concentrations (10, 20, 30, 40, 50 µg/mL) of both amla and acerola extracts were separately added to 200µL of the DPPH working solution in individual wells of a 96-well plate. The plate was then incubated in darkness for 30 minutes at room temperature. Following the incubation period, the absorbance of each well was measured at 517 nm using a microplate reader, with methanol serving as the blank. The percentage of DPPH scavenging activity was determined using the formula:

%DPPH Scavenging Activity = (Acontrol - Asample/Acontrol) x 100

Where A control represents the absorbance of the control (DPPH solution without the sample) and A sample represents the absorbance of the sample (DPPH solution with either amla or acerola extract). The positive control group included ascorbic acid at a concentration of 1 mg/mL.

## Anti-inflammatory activity:

The comparative anti-inflammatory activity of both amla and acerola extract was assessed using the Bovine Serum Albumin (BSA) denaturation assay. In this assay, 0.45mL of bovine serum albumin was combined with 0.05 mL of different concentrations (10-50 µg/mL) of both amla and acerola extracts. The pH of the mixture was adjusted to 6.3, and the solution was allowed to stand at room temperature for 10 minutes. Subsequently, the samples were incubated in a water bath at 55°C for 30 minutes. Diclofenac sodium served as the standard group, while dimethyl sulfoxide (DMSO) was employed as the control. Following the incubation period, the samples were spectrophotometrically measured at 660nm. The degree of inhibition of BSA denaturation by the amla and acerola extracts was determined. The results were compared with the standard diclofenac sodium and dimethyl sulphoxide as control. Then, the samples were measured spectrophotometrically at 660nm. Percentage of protein denaturation was determined utilizing following equation,

% inhibition = Absorbance of control - Absorbance of sample x 100 Absorbance of contro1

## Antimicrobial activity:

The antimicrobial activity of amla and acerola extracts was assessed using the agar well diffusion technique. Mueller Hinton agar plates were sterilized and inoculated with bacterial suspensions of *Staphylococcus aureus*, *Pseudomonas aeruginosa*, and *Escherichia coli*. Wells were created in the agar, filled with varying concentrations (25 µL, 50 µL, 100 µL) of both extracts, while amoxyrite served as a standard. After incubation at 37°C for 24 hours, the inhibition zones around the wells were measured using a ruler. The recorded values were then utilized to calculate and compare the antibacterial efficacy of amla and acerola extracts with the standard antibiotic, providing insights into their potential inhibitory effects on bacterial growth.

## Cytotoxic effect:

The Brine shrimp lethality assay was employed to compare the cytotoxic effects of amla and acerola extracts. Saline water was prepared by dissolving 2 grams of iodine-free salt in 200 mL of distilled water, and 10 to 12 mL of this solution was added to each well of six-well ELISA plates. Subsequently, 10 nauplii were gently introduced into each well, followed by the addition of various concentrations of both amla and acerola extracts. The loaded plates were then incubated at room temperature for 24 hours. After this incubation period, the ELISA well plates were examined, and the count of live nauplii was recorded. The cytotoxicity was calculated using the formula:

Number of dead nauplii / (Number of dead nauplii + Number of live nauplii) x 100

## Results and Discussion:

## Anti-inflammatory activity:

**Figure 1:**
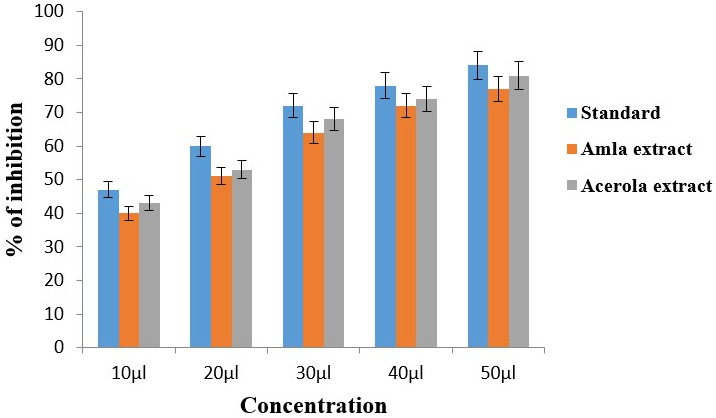
Anti-inflammatory activity of amla and acerola extract using Bovine serum albumin denaturation assay

Bovine serum albumin denaturation assay:

In Figure 1, the anti-inflammatory effects of Amla and Acerola extracts were evaluated across concentrations (10µl to 50µl) in comparison with standard diclofenac sodium. At 10µl, both extracts exhibited lower anti-inflammatory responses (40% for Amla, 43% for Acerola extract) compared to the standard (47%), with Acerola demonstrating a slightly heightened effect. This trend continued as concentrations increased: at 20µl, Amla extract reached 51%, Acerola extract 53%, and the standard 60%; at 30µl, Amla extract reached 64% of inhibition, Acerola extract around 68%, and the standard at 72%; at 40µl, Amla and Acerola extract recorded values of 72% and 74%, respectively, below the standard (78%); and at 50µl, Amla extract reached 77%, Acerola extract at 81%, both below the standard (84%). Acerola extract consistently outperformed amla extract across all concentrations, indicating a sustained concentration-dependent enhancement of anti-inflammatory properties. These findings suggest that acerola extract may serve as a more potent anti-inflammatory agent compared to both amla extract and the standard across the concentration spectrum studied.

## Antioxidant activity:

**Figure 2:**
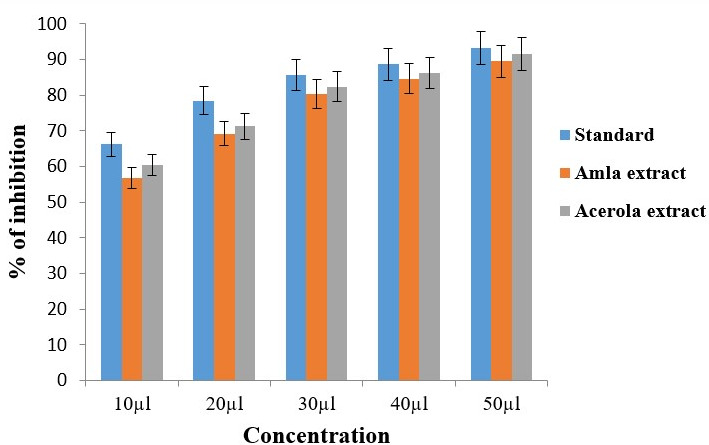
Antioxidant activity of amla and acerola extract tested by adopting DPPH assay

DPPH assay:

The study investigated the concentration-dependent effects of Amla and Acerola extracts using DPPH assay (Figure 2) across a range of concentrations (10µl to 50µl). Starting at 56.8% for 10µl, the concentration-dependent response of Amla extract was evident. The values increased gradually, reaching 89.58% at 50µl.

Similarly, Acerola extract exhibited a concentration-dependent effect. The values started at 60.39% for 10µl and progressively increased to 91.62% at 50µl. The consistent rise in values indicates that Acerola extract, like Amla extract, influences the antioxidant effect in a concentration-dependent manner. At each concentration point, Amla extract consistently displayed lower values than Acerola extract. This discrepancy suggests that, at equivalent concentrations, Acerola extract may have a more potent antioxidant effect compared to Amla extract. Moreover, the rate of increase in values varied between the two extracts. While Amla extract exhibited a relatively steady progression, Acerola extract demonstrated a slightly steeper increase. This difference in the slopes of the concentration-response curves implies that Acerola extract may have a faster and more pronounced impact as the concentration increases.

## Antibacterial activity:

**Figure 3:**
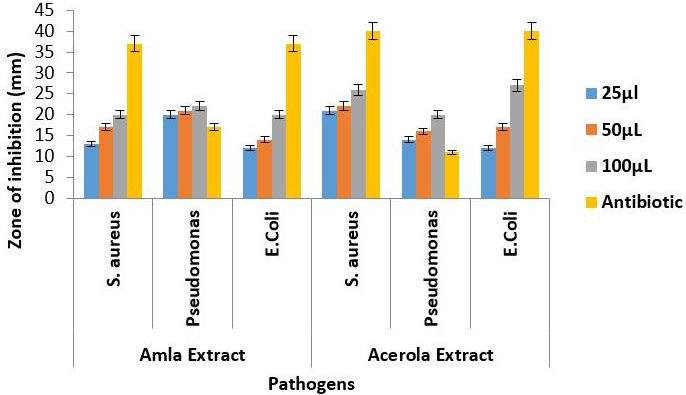
Antibacterial activity of amla and acerola extract against oral pathogens tested by using agar well diffusion technique

Agar well diffusion technique:

The amla extract (Figure 3) demonstrated a concentration-dependent antibacterial effect against *Staphylococcus aureus*. At concentrations of 25µl, 50µl, and 100µl, the zones of inhibition were measured at 13mm, 17mm, and 20mm, respectively. The increase in the zone of inhibition suggests that higher concentrations of amla extract correlate with enhanced suppression of *Staphylococcus aureus* growth. Amla extract exhibited significant inhibitory activity against *Pseudomonas*. The zones of inhibition were observed at 20mm, 21mm, and 22mm for concentrations of 25µl, 50µl, and 100µl, respectively. This indicates a progressive increase in antibacterial efficacy with higher concentrations of amla extract. Against *Escherichia coli*, amla extract demonstrated pronounced antibacterial effects. Zones of inhibition were measured at 12mm, 14mm, and 20mm for concentrations of 25µl, 50µl, and 100µl, respectively. The results suggest that amla extract has a notable impact on inhibiting the growth of *Escherichia coli*, with increasing potency at higher concentrations.

Acerola extract (Figure 3) displayed antibacterial activity against *Staphylococcus aureus*. The zones of inhibition were 21mm, 22mm, and 26mm for concentrations of 25µl, 50µl, and 100µl, respectively. The increasing trend in the zones of inhibition indicates a dose-dependent response, suggesting a potential concentration-related enhancement of antibacterial properties. Similar to its impact on *Staphylococcus aureus*, acerola extract exhibited inhibitory effects against *Pseudomonas*. Zones of inhibition measured at 14mm, 16mm, and 20mm for concentrations of 25µl, 50µl, and 100µl, respectively. The results suggest that acerola extract possesses significant antibacterial activity against *Pseudomonas*, with higher concentrations leading to larger zones of inhibition. Acerola extract also showcased remarkable antibacterial efficacy against *Escherichia coli*. Zones of inhibition were measured at 12mm, 17mm, and 27mm for concentrations of 25µl, 50µl, and 100µl, respectively. The concentration-dependent increase in the zones of inhibition suggests a potent antibacterial effect, with higher concentrations of acerola extract exhibiting greater inhibitory activity against *Escherichia coli*.

Amoxyrite, the tested antibiotic, exhibited significant antibacterial activity against the selected oral pathogens. The antibiotic tested against *Staphylococcus aureus*, the zones of inhibition remained consistently substantial, measuring 37mm, 40mm, and 37mm at concentrations of 25µl, 50µl, and 100µl, respectively. Amoxyrite displayed varying inhibitory effects with zones of 17mm, 11mm, and 40mm at concentrations of 25µl, 50µl, and 100µl, against *Pseudomonas*, suggesting a concentration-dependent response. Particularly noteworthy was its antibacterial activity against *Escherichia coli*, maintaining substantial zones of inhibition at 37mm, 40mm, and 37mm for concentrations of 25µl, 50µl, and 100µl.

## Cytotoxic effect:

**Figure 4:**
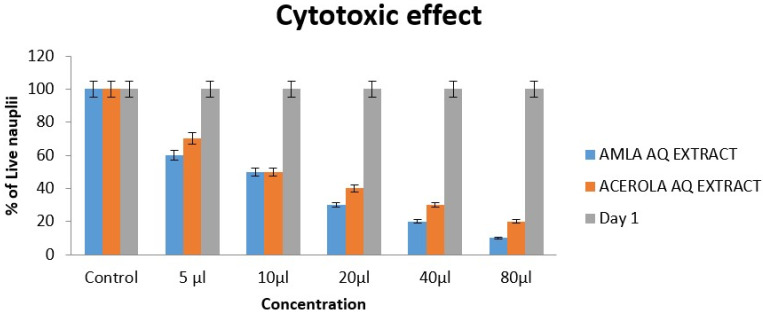
The cytotoxic effect of amla and acerola extract using Brine shrimp lethality assay

Brine shrimp lethality assay:

In the brine shrimp lethality assay, the cytotoxic effects of Amla and Acerola aqueous (AQ) extracts (Figure 4) were systematically evaluated across various concentrations (5 µl to 80 µl). The control group exhibited 100% viability on both Day 1 and Day 2, establishing a baseline for comparison. Notably, Amla extract displayed a more pronounced reduction in brine shrimp nauplii viability compared to Acerola extract. At the highest concentration (80 µl), amla extract resulted in a substantial 90% reduction in live nauplii, underscoring a potent dose-dependent cytotoxic effect. Conversely, acerola extract exhibited an 80% reduction at the same concentration. These findings suggest a differential cytotoxic impact of the two extracts, indicating the need for further exploration of their specific chemical constituents and potential applications in biomedical research.

## Discussion:

The presented study investigated the comparative antioxidant, anti-inflammatory, and antimicrobial activities of aqueous extracts derived from Acerola and Amla. In the anti-inflammatory potential of both extracts using the bovine serum albumin denaturation assay, Acerola consistently outperformed Amla across concentrations, suggesting a sustained concentration-dependent enhancement of anti-inflammatory properties. The results reveal that Acerola extract may serve as a more potent anti-inflammatory agent compared to both Amla extract and the standard diclofenac sodium [21]. DPPH assay was employed to assess the antioxidant activities of Amla and Acerola extracts. Both extracts demonstrated concentration-dependent antioxidant effects, with Acerola consistently exhibiting higher values than amla at equivalent concentrations. The study indicates that Acerola extract may possess a more potent antioxidant effect compared to Amla extract across the concentration spectrum studied. Moreover, the rate of increase in antioxidant values varied, with Acerola extract showing a slightly steeper and faster progression, suggesting a potentially quicker and more pronounced impact as the concentration increases [22]. In Agar well diffusion method for antibacterial activities of Amla and Acerola extracts against oral pathogens, Amla extract displayed concentration-dependent antibacterial effects against *Staphylococcus aureus*, *Pseudomonas*, and *Escherichia coli*. Acerola extract consistently demonstrated larger zones of inhibition, indicating a potential concentration-related enhancement of antibacterial properties. The findings suggest that Acerola extract may be a more potent antibacterial agent compared to Amla extract across various concentrations [23]. The results on cytotoxic effects of Amla and Acerola assessed using the brine shrimp lethality assay, indicate a dose-dependent cytotoxic effect for both extracts, with Amla exhibiting a more pronounced reduction in brine shrimp nauplii viability compared to Acerola. This differential cytotoxic impact emphasizes the need for further exploration of the specific chemical constituents within the extracts and their potential applications in biomedical research. The aqueous extracts of Acerola and Amla have been identified as rich sources of potent antioxidants, anti-inflammatory agents, and antimicrobial compounds. Acerola extracts, abundant in ascorbic acid and phenolic compounds, exhibit significant antioxidant activity along with some antimicrobial effects [21]. Similarly, Amla extracts, characterized by high levels of vitamin C and polyphenols, act as robust antioxidants, inhibiting lipid peroxidation and preserving antioxidant enzyme activity [24]. The antioxidant prowess of Acerola extracts is attributed to their elevated vitamin C content, total phenol index, and polyphenolic compounds [25]. Amla extracts have demonstrated additional anti-collagenase and anti-elastase activities, reinforcing their anti-inflammatory properties [26]. These research findings collectively underscore the significant potential of Acerola and Amla extracts as natural reservoirs of antioxidants and antimicrobial agents, offering promising avenues for further exploration in the fields of health and medicine. Overall, this comprehensive analysis suggests that Acerola extract generally outperforms Amla extract in terms of anti-inflammatory, antioxidant, and antibacterial activities. However, the differential impact on cytotoxicity raises important considerations for the overall safety and potential therapeutic applications of these extracts.

## Conclusion:

Data shows that acerola extract consistently outperforms Amla across multiple assays, showcasing its efficacy as a potent anti-inflammatory and antioxidant agent. The superior antibacterial activities of Acerola, particularly against prevalent oral pathogens, underline its potential for therapeutic applications in combating bacterial infections. Ultimately, while Acerola emerges as a promising source for multifaceted pharmacological benefits, the detailed interplay between efficacy and cytotoxicity prompts a balanced consideration of its applications.
